# Gut Microbiota Modulation: A Novel Strategy for Rheumatoid Arthritis Therapy

**DOI:** 10.3390/biom14121653

**Published:** 2024-12-23

**Authors:** Vitaly Chasov, Elvina Gilyazova, Irina Ganeeva, Ekaterina Zmievskaya, Damir Davletshin, Aygul Valiullina, Emil Bulatov

**Affiliations:** 1 Laboratory of Biomedical Technologies, Institute of Fundamental Medicine and Biology, Kazan Federal University, 18 Kremlyovskaya Street, Kazan 420008, Russiakovirka1995@gmail.com (I.G.);; 2 Shemyakin-Ovchinnikov Institute of Bioorganic Chemistry, Russian Academy of Sciences, Moscow 117997, Russia

**Keywords:** rheumatoid arthritis, gut microbiota, dysbiosis, bacterial translocation, molecular mimicry, bacterial metabolites

## Abstract

Rheumatoid arthritis (RA) is a chronic autoimmune disease that leads to joint inflammation, progressive tissue damage and significant disability, severely impacting patients’ quality of life. While the exact mechanisms underlying RA remain elusive, growing evidence suggests a strong link between intestinal microbiota dysbiosis and the disease’s development and progression. Differences in microbial composition between healthy individuals and RA patients point to the role of gut microbiota in modulating immune responses and promoting inflammation. Therapies targeting microbiota restoration have demonstrated promise in improving treatment efficacy, enhancing patient outcomes and slowing disease progression. However, the complex interplay between gut microbiota and autoimmune pathways in RA requires further investigation to establish causative relationships and mechanisms. Here, we review the current understanding of the gut microbiota’s role in RA pathogenesis and its potential as a therapeutic target.

## 1. Introduction

Rheumatoid arthritis (RA) is an autoimmune disease characterized by chronic inflammation of the synovial joints, primarily small peripheral joints, that can lead to heart, lung, or nervous system disorders, the impairment of other vital body functions and severe disability [1]. The disease affects approximately 1% of adults worldwide, predominantly women [2]. The aetiology of RA is complex and not fully understood [3]. It is believed that the autoimmune response is triggered by a combination of genetic and environmental factors [4]. Genetic predisposition plays an important role, as a dramatically increased risk of RA has been identified in individuals who carry certain genes, and first-degree relatives of RA patients are more likely to develop the disease than the general population [5,6,7]. Among environmental factors, smoking is the most important risk factor for developing RA [8]. RA has a female to male susceptibility ratio of 3:1, suggesting that female sex hormones also influence the development of the disease [9]. This is supported by the inverse correlation between RA severity and androgen levels [10]. There is also evidence that while testosterone and progesterone naturally suppress the immune system, oestrogens, especially 17-β oestradiol (E2) and prolactin, act as enhancers of at least humoral immunity [11,12]. It is also important to note that during pregnancy, E2 has mainly anti-inflammatory effects by inhibiting the production and signalling of pro-inflammatory cytokines [13,14].

The known pathogenesis of RA includes the production of autoantibodies mediated by aberrantly activated lymphocytes, the activation of inflammatory pathways and synovial proliferation [15]. In the synovial tissue of some patients, immune cells diffusely infiltrate the synovial membrane, while in others T cells and B cells cluster into aggregates with or without follicular dendritic cells (DCs), forming lymphoid aggregates or ectopic germinal centres (GCs), leading to a breakdown of self-tolerance and acceleration of RA pathogenesis [16]. T cells are the dominant lymphocytes infiltrating the joints of rheumatoid patients, with a predominance of CD4+ T cells over CD8+ T cells in the majority of patients [17]. Activated B cells produce autoantibodies such as rheumatoid factor (RF) and anti-citrullinated protein antibodies (ACPA) and generate further inflammation [18]. RF and ACPA are diagnostic markers for RA, and RA patients are classified as seropositive or seronegative depending on the presence or absence of these antibodies [19]. T cells activate macrophages, leading to an overproduction of inflammatory cytokines [20]. An imbalance in the ratio of anti-inflammatory to pro-inflammatory cytokines is determined by changes in the Th1/Th17 cell profile [7,21]. Macrophages exert their effects through phagocytosis, the production of antigen presenting cells (APCs), additional pro-inflammatory cytokines (IL-1β, IL-6, TNF-α) and matrix-degrading enzymes [7]. IFN-γ is also an important cytokine in the pathology of RA that contributes to the establishment of early inflammation in RA [7]. The increased production of cytokines promotes the proliferation of synovial fibroblasts and the expansion of the synovial membrane, pannus, which can affect bone and cartilage and lead to joint destruction [7]. Synovial fibroblasts have been shown to be the major source of the receptor activator of NF-κB ligand (RANKL), which is primarily responsible for osteoclast differentiation and subsequent bone erosion [22,23]. Osteocytes have also been found to play an important role in inflammatory bone resorption, as TNF-α directly affects RANKL expression in osteocytes and promotes osteoclast formation [24]. During the inflammatory processes in the joints of RA patients, the expansion of the synovial tissue and the associated hypoxia require a compensatory increase in the number and density of synovial blood vessels, driving the formation of new blood vessels, known as angiogenesis [25]. Angiogenesis has been shown to play an important role in the pathogenesis of RA, increasing cartilage erosion and pannus formation [26].

In 2010, the European League Against Rheumatism (EULAR) developed recommendations for the treatment of RA with drugs, and these recommendations have been updated every 3 years since then [27]. Current treatments for RA include non-steroidal anti-inflammatory drugs (NSAIDs), immunosuppressive steroids (glucocorticoids) and disease-modifying antirheumatic drugs (DMARDs) [7]. NSAIDs are usually used only for symptomatic treatment to improve joint function and reduce pain and swelling, but are not disease-modifying as they do not prevent further joint damage and can be toxic [28,29]. Glucocorticoids such as prednisolone are non-specific immunosuppressants with disease-modifying effects in early RA, but their long-term use is limited due to severe multisystemic metabolic side effects such as gastrointestinal bleeding, osteoporosis and ulceration [7,29]. There are currently several subclasses of DMARDs. One of these is the conventional synthetic DMARDs (csDMARDs), which include the most commonly used, methotrexate, hydrochloroquine and sulfadiazine. Although this group of drugs can help patients achieve either remission or low disease activity, there are still many patients who do not respond to csDMARDs [30]. csDMARDs are also associated with the frequent occurrence of side effects such as liver damage, cytopenia, rash, fatigue, nausea, stomatitis and hair loss [7]. In recent decades, two other subclasses of DMARDs have been developed that act on a specific set of molecules involved in the inflammatory process. Targeted synthetic drugs (tsDMARDs) include synthetic molecules such as Janus kinase (JAK) inhibitors, which can effectively inhibit the progression of RA and significantly improve the prognosis of RA, but their long-term use can cause side effects [7]. For example, the application of JAK inhibitors is frequently accompanied by the formation of blood clots, an elevation of blood cholesterol levels, cytopenia, an increased frequency of infections (often with Herpes zoster) and gastrointestinal diseases [31,32]. Although biologic DMARDs (bDMARDs) such as B-cell depleting antibodies, TNF-α inhibitors (soluble TNF receptor and monoclonal antibodies (mAbs) to TNF-α), IL-6 inhibitors (mAbs to IL-6 receptor) and co-stimulatory molecule inhibitors (soluble CTLA-4-Fc) have demonstrated the potential to significantly improve symptoms and prevent disease progression in RA patients, they are immunogenic and can increase the risk of serious infections [2]. Another serious disadvantage of biologics is their high cost [33]. It is important to note that according to the most recent updates of the EULAR recommendations, bDMARDs and tsDMARDs should be combined with a csDMARD, with bDMARDs being preferred over JAK inhibitors because of their higher cardiovascular risk factors and higher malignancy rates, although prior to 2019, JAK inhibitors were considered to be similar to bDMARDs in terms of efficacy and safety [34].

Due to the lack of efficacy and serious side effects of existing methods for the treatment of RA, it is an urgent task to search for and develop new approaches that are affordable, efficient and safe. Therefore, the search for new ways to treat RA and new targets for drug action continues. In this regard, the set of microorganisms inhabiting the gut, the microbiota, is a suitable object of study. It is in the gastrointestinal tract that the majority of human microbiota are found, accounting for 97% of the total, with the main habitat being the large intestine [35]. Since the gut microbiota has been shown to mediate a wide range of physiological functions, contribute to the maintenance of immunological homeostasis, serve as a gauge of host health, and any disruption in its composition can lead to the development of disease, it is not surprising that dysbiosis of the gut microbiota has been implicated in the development of many systemic autoimmune diseases (SAIDs), including RA [36]. It was also demonstrated that some success in treating RA can be achieved by manipulating the gut microbiota [36]. Correcting the gut microbiota can be the extra drop that tips the scales toward the patient’s recovery. This review is dedicated to describing the role of the gut microbiota in the pathogenesis of RA and treatment strategies that involve influencing and modifying the microbiota to eliminate its dysbiosis.

## 2. The Connection Between Gut Microbiota Dysbiosis, Immune System Dysregulation and RA Progression

The human microbiota is a diverse and dynamic collection of microorganisms, including bacteria, fungi and viruses, with their genetic material and waste products. It is difficult to establish universal biomarkers of what constitutes a healthy microbiota, as metagenomic analysis of faecal samples from individuals in four countries identified three groups (enterotypes) of gut microbiota that are independent of nationality and differ in the predominant species of microorganisms [37]. In addition, the individuality of the gut microbiota is determined by other factors, including diet, genetics and environmental exposures [38]. In general, however, a healthy or balanced microbiota is characterised by the following features. First, a high diversity of bacteria, i.e., a large number of different species inhabiting the intestine. The second characteristic is the specific composition of the microbiota, in which the species of bacteria that are considered beneficial predominate over those that are harmful or opportunistic. However, this characteristic cannot be considered universal, as some species that are generally considered beneficial may be harmful under certain conditions and, conversely, the presence of potentially pathogenic microorganisms does not always correlate with disease. The ability of the microbiota to maintain certain functions is another sign of its health. These include supporting the gut’s barrier function to prevent the invasion of potential environmental pathogens, assisting in the digestion of food components, the biosynthesis of vitamins and bioactive metabolites, and supporting the tolerance of the immune system [38]. A disruption of these three hallmarks of a healthy microbiome is considered dysbiosis, which is characteristic of any disease.

When the balance of gut microbiota is disrupted, it can affect mucosal and systemic immunity and promote RA through the activation of the so-called “gut–joint axis” [39,40]. The dysbiosis of the gut microbiota has been found in various stages of RA, and in addition, many data suggest a relationship between microbiota characteristics and immune dysfunction in RA. Changes in microbiota composition and pro-inflammatory cytokine levels in RA according to existing data are summarised in the table below (Table 1). In particular, several animal studies have shown that changes in the gut microbiota can affect local and systemic immunity, leading to joint inflammation [41,42]. *Faecalibacterium prausnitzii* is one of the important commensal bacteria in the human gut microbiota, accounting for 3–5% of the total number of bacteria detected in stool samples of healthy people [43]. The anti-inflammatory effects of this bacterium were demonstrated in a mouse model of RA [44]. The administration of *F. prausnitzii* to collagen-induced arthritis (CIA) mice was found to exert a therapeutic effect on RA by regulating IL-17 production [44]. The conventionalisation of germ-free mice with the microbiota of CIA-susceptible mice has been shown, in another study, to significantly increase the abundance of Th17 cells and reduce the proportion of Treg cells compared to those conventionalised with the microbiota of CIA-resistant mice, suggesting that the gut microbiota profoundly affects the balance between pro- and anti-inflammatory immune responses [45]. In addition, the genera *Desulfovibrio*, *Prevotella*, *Parabacteroides*, *Odoribacter*, *Acetatifactor*, *Blautia*, *Coprococcus* and *Ruminococcus* were abundant, and levels of serum IL-17 and splenic CD4+ Th17 cells were elevated in arthritic mice, suggesting that the gut microbiota composition differs between CIA-susceptible and CIA-resistant mice [45]. At the same time, antibiotic treatment worsened arthritis in CIA mice and increased levels of pro-inflammatory cytokines such as interleukin (IL)-6, interferon (IFN)-gamma and IL-17 [45]. It was also found that the abundance of the phylum *Bacteroidetes*, specifically families *S24-7* and *Bacteroidaceae*, was reduced, whereas *Firmicutes* and *Proteobacteria*, such as *Ruminococcaceae*, *Lachnospiraceae* and *Desulfovibrinocaceae*, were expanded during the initiation phase of arthritis in CIA mice [46]. In addition, the elimination of the gut microbiota during established arthritis specifically reduced gut Th17 cells and attenuated arthritis, suggesting that gut microbiota perturbations precede arthritis and that modulating the gut microbiota after the onset of arthritis may offer therapeutic opportunities [46]. In one of the studies, faecal samples from RA patients were inoculated into germ-free arthritis-prone SKG mice and the immune responses were assessed [47]. Gut dysbiosis was shown to increase susceptibility to arthritis via the activation of autoreactive T-cells, causing joint inflammation [47].

Findings in mouse models have been confirmed in studies of RA patients. 16S ribosomal DNA sequencing analysis revealed decreased bacterial diversity and Actinobacteria expansion in RA patients compared to healthy controls [48]. Three genera were also associated with RA, specifically an increase in *Collinsella* and *Eggerthella* and a decrease in *Faecalibacterium* [48]. An examination of the gut microbiota of RA patients with positive anti-cyclic citrullinated peptide (anti-CCP) antibodies with different clinical characteristics using metagenomic analysis also revealed reduced microbial diversity and a higher abundance of the genera *Verrucomicrobiae*, *Blautia*, *Akkermansia and Clostridiales* compared to healthy controls [49]. In addition, patients with high TNFα and IL-17A levels showed an increase in *Enterobacteriaceae* and *Klebsiella* and a decrease in *Bifidobacterium*, while ACPA-positive patients showed an increase in *Blautia*, *Akkermansia* and *Clostridiales* [49]. A study performed by the same method showed an increased abundance of *Prevotellaceae* (*Prevotella denticola*) in the gut of RA patients [50]. A comparison of 110 RA patients with the same number of healthy controls showed that the abundance of *Collinsella* and *Bifidobacterium* was increased in RA patients [51]. The metabolic profile of the gut microbiota was also characterized by differing pathways [51]. The next study showed an increase in Collinsella aerofaciens, Sedimentibacter and Enterococcus genera in patients and a decrease in Dorea formicigenerans compared to the control group [52]. Since *Enterococci* are pathobionts, it is suggested that a shift in the ratio of symbionts to pathobionts from the gut microbiota could create an inflammatory imbalance and induce Th17 or Th1 responses [52]. Lactobacteria were also found to be significantly decreased in RA patients, whereas *Enterococci* and *Clostridia* were significantly increased [53]. Furthermore, the proportion of *Bifidobacteria*, *Bacteroids* and *Lactopositive colibacteria* was observed to be reduced, while the abundance of opportunistic *Enterobacteria* and *Staphylococci* was increased [53].

The study involving patients with an early diagnosis of RA (up to 6 months after the diagnosis) found an increase in the *Prevotella* species and a decrease in *Bacteroides* species [54]. In another study, patients with newly diagnosed RA with anti-CCP antibodies were also found to have a distinct microbiome compared to healthy controls [55]. This group of patients had increased levels of *Lachnospiraceae*, *Helicobacteraceae*, *Ruminococcaceae*, *Erysipelotrichaceae* and *Bifidobacteriaceae* in their intestines [55].

It has been found that the composition of the microbiota changes in RA not only in the gut, but also in the mouth. Microbiota in faecal, dental and salivary samples from RA patients was analysed using metagenomic sequencing and a metagenome-wide association study [56]. The results showed that *Haemophilus* spp. were depleted, while *Lactobacillus salivarius* was increased in the gut of RA patients [56]. Autoantibody levels (both rheumatoid factor (RF) and ACPA) were also associated with a decrease in *Haemophilus* spp., while higher inflammatory protein levels were associated with an increase in *Lactobacilus salivaries.* Similar findings were observed in their dental and salivary samples [56]. It should be noted that the available data suggest the existence of a link between the gut–oral microbiota and joint inflammation in RA, the so-called oral–gut axis [57,58,59].

## 3. Mechanisms That Link Gut Microbiota Dysbiosis and RA Pathogenesis

**Figure 1:**
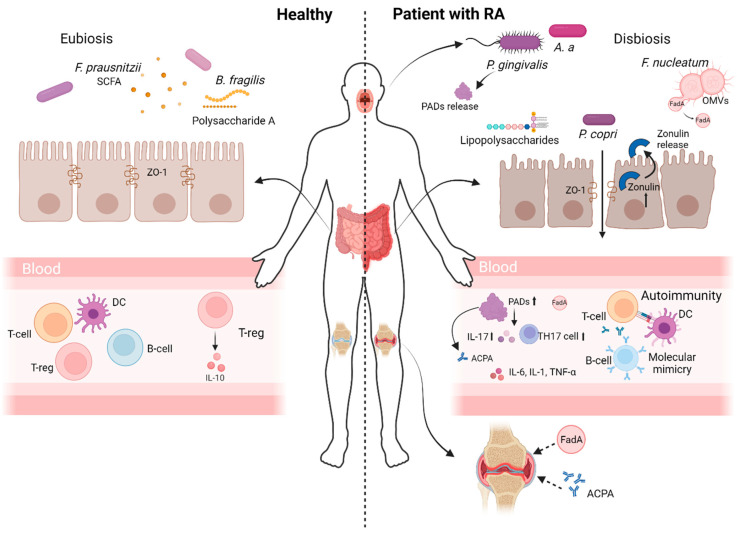
Mechanisms linking oral and gut microbiota dysbiosis to RA pathogenesis. The figure illustrates the contrasting states of a healthy individual and a patient with rheumatoid arthritis (RA). Healthy state (**left panel**): A balanced gut microbiota ensures a robust intestinal barrier regulated by tight junction molecules like ZO-1. Beneficial microbes such as *Faecalibacterium prausnitzii* and *Bacteroides fragilis* produce short-chain fatty acids (SCFAs) and polysaccharide A, which support immune tolerance by promoting Treg differentiation, balancing Th1/Th17 responses, and producing anti-inflammatory cytokines (e.g., IL-10). RA state (**right panel**): Dysbiosis leads to increased intestinal permeability via zonulin release and compromised tight junctions, facilitating bacterial translocation. Pathogenic microbes, including *Prevotella copri* and *Fusobacterium nucleatum*, interact with immune cells (B cells, T cells and dendritic cells) to induce inflammatory responses, producing autoantibodies (ACPA and RF) and inflammatory cytokines (IL-6, IL-1 and TNF-α). Molecular mimicry by bacterial proteins further exacerbates autoimmunity. Oral pathogens like *Porphyromonas gingivalis* contribute to ACPA formation via PAD enzymes and heightened pro-inflammatory cytokines through lipopolysaccharides (LPSs). Virulence factors such as FadA from *F. nucleatum* drive joint inflammation through outer membrane vesicles (OMVs).

Evidence of the strong correlation between gut microbiota dysbiosis and RA pathogenesis suggests several mechanisms to explain the possible role of the microbiota in the pathogenesis of RA, but most of them require further elucidation. These mechanisms are currently thought to be bacterial translocation due to intestinal permeability, molecular mimicry and the production of metabolites that may cause citrullination or have immunomodulatory properties (Figure 1).

### 3.1. Bacterial Translocation

One of the mechanisms by which the microbiota may contribute to the development of RA is through microbial translocation (Figure 1) [60]. An intact intestinal barrier is essential to separate the intestinal epithelium from toxins, microorganisms and antigens in the intestinal lumen. The maintenance of intestinal barrier function is known to be regulated by zonulin, a protein whose levels are elevated in patients with established RA [61]. Moreover, the treatment of CIA mice with larazotide acetate, which directly blocks zonulin, prior to the onset of arthritis prevented the observed increase in intestinal barrier permeability and attenuated arthritis symptoms [61]. This experiment demonstrates that the disruption of the intestinal barrier plays an important role in the pathogenesis of RA. Imbalances in the gut microbiota are likely to lead to alterations in immune homeostasis, resulting in a breach of the intestinal barrier [62]. These changes allow microbes and their metabolites to invade the lamina propria and subepithelial spaces, and microbial antigens may activate autoreactive B and T cells, which are associated with the risk and severity of RA [63].

Several experimental data suggest increased intestinal permeability during RA. Because opportunistic *Enterobacteriaceae* have been detected in the urine and nasal mucosa of RA patients, it has been suggested that Enterobacteriaceae may translocate from the gut to other organs [53]. Bacterial nucleic acids have also been detected in the synovial fluid of RA patients [64]. 16S ribosomal DNA sequencing of faecal samples from RA patients revealed increased levels of *Collinsella* [48]. Changes in intestinal permeability were determined using 4-KDa FITC-labelled dextran, and *Collinsella* was found to increase intestinal permeability by reducing the expression of tight junction protein ZO-1 in epithelial cells in a mouse model [48]. The recently discovered *S. didolesgii isolate* 7 was also shown to have increased access to the host intestinal epithelium, where it induced the formation of large isolated lymphoid follicles [65]. Taken together, these studies provide evidence that different species of bacteria can penetrate the intestinal wall and induce an immune response. Further studies are needed to understand in detail how specific microbes cross the barrier.

### 3.2. Molecular Mimicry

Bacterial products and antigens could also stimulate antibodies against autoantigens via antigenic mimicry [66]. Molecular mimicry is a condition, in which molecular structures of a microorganism resemble those of the host [67]. The bacterial species with the most evidence for its ability to induce autoimmunity through molecular mimicry is *P. copri*, although not everything about this mechanism is fully understood (Figure 1). The expansion of *P. copri* in patients with new-onset RA was firstly demonstrated [54]. In addition, coculturing SKG dendritic cells with *Prevotella copri* has been found to activate autoreactive cells in the gut and exacerbate joint inflammation in response to RA autoantigens [47]. A study of faecal samples in patients with RA performed by 16S rRNA sequencing also indicated that gut dysbiosis is associated with *Prevotella copri* [47,54]. The causal role of *Prevotella copri* in the progression of RA has also been confirmed, since the study of stool samples from 133 individuals in preclinical stages of RA also revealed a significant enrichment of the bacterial family *Prevotellaceae*, particularly *Prevotella*, compared with healthy controls [68]. To clarify the mechanism of influence of *Prevotella copri* in RA pathogenesis, the antigenicity of peptide derived from this bacteria was examined in samples from the RA patients [69]. For this study, a 27-kd protein of *P. copri* (Pc-p27) was used that has minimal sequence homology with any human peptide and stimulated Th1 responses in 42% of patients with new-onset RA [69]. Patients with RA were found to have anti-Pc-p27 antibody responses that correlated with ACPA, which were rarely found in patients with other rheumatic diseases or in healthy individuals [69]. The next study, performed by the same authors, identified, in the synovial tissue of RA patients, HLA-DR-presented self-antigens, Filamin A and N-acetylglucosamine-6-sulfatase, that have considerable sequence homology with *Prevotella* epitopes and with similar epitopes from several related gut commensals [70]. T cell responses to the corresponding microbial and self-peptides were shown to be strongly correlated, suggesting that T cell epitope mimicry may provide a potential link between mucosal immunity and immune responses in affected joints and cross-reactivity may occur between human and bacterial epitopes [70]. Then, five additional HLA-DR-presented neoepitopes from *P. copri* were identified from three patients with RA [71]. In their cohort, 74% of RA patients exhibited T cell responses and 53% exhibited IgG or IgA reactivity to at least one of the epitopes [71]. Furthermore, anti-*P. copri* IgA antibody titres positively correlated with anti-citrullinated protein antibody (ACPA) levels, suggesting that antibody responses to *P. copri* neoepitopes may contribute to the ACPA response [71].

TCR cross-reactivity has also been observed between vinculin, which is expressed in the synovium and can be citrullinated, and bacterial antigens [72]. IgG and IgA autoantibodies from individuals at risk for RA have recently been shown to cross-react with gut bacteria from the *Lachnospiraceae* and *Ruminococcaceae* families. These antibody-bound bacteria were found to be able to induce T-cell responses in patients with RA, as well as arthritis in mice [65]. Taken together, these findings suggest that gut bacteria may act as molecular mimics that trigger autoimmune responses in RA patients.

### 3.3. Bacterial Metabolites

#### 3.3.1. Metabolites That May Cause Citrullination

There are two possible ways in which bacterial metabolites may influence the pathogenesis of RA. Some substances produced by bacteria are associated with the mechanism of ACPA formation in RA (Figure 1). The detection of 21 citrullinated peptides in the colon tissue of RA patients suggests both an impairment of intestinal barrier function and that the presence of citrullinated antigens may trigger immune responses in RA [73]. For example, *Eggerthella lenta*, a bacterium that uses ornithine as an energy substrate and produces carbamoyl phosphate and citrulline as by-products, is found in large numbers in the intestines of RA patients [48]. It is hypothesized that the high abundance of *Eggerthella* in the gut of RA patients produces increased levels of citrulline available for citrullination, against which antibodies may be produced [48]. Recent studies have revealed that oral bacteria present in saliva can colonize the intestinal tract and become an integral component of the gut microbiota [74,75,76]. In addition, the bacteria that cause periodontal disease (PD) are thought to cause dysbiosis and the disruption of intestinal barrier function, as well as alterations in bacterial metabolites and the disruption of immune tolerance [59]. Specific oral pathogens, such as *Porphyromonas gingivalis*, *Prevotella intermedia* and *Fusobacterium nucleatum*, have been postulated as a link between PD and RA [77,78,79]. Several studies report that citrullination in RA can be induced by *P. gingivalis* and *Aggregatibacter actinomycetemcomitans* (*Aa*), which is another pathobiont of PD [80,81,82]. Citrullination occurs when arginine residues are converted to citrulline residues by a secreted membrane-associated peptidyl-arginine deiminase, a group of enzymes called PAD, which catalyse the hydrolysis of peptidyl-arginine to peptidyl-citrulline and causes the recognition of amino acid chains as autoantigens, resulting in the production of ACPA [62]. *P. gingivalis* was shown to produce PAD (Figure 1) [83]. PAD from *P. gingivalis* can citrullinate endogenous proteins, including human RA-relevant autoantigens fibrinogen and α-enolase [84]. *Aa* produces leukotoxin A, a pore-forming virulence factor that induces hypercitrullination in neutrophils [85]. In addition, an analysis of citrullinated bacterial epitopes in the saliva of patients with periodontal disease showed that *Aa* itself can produce cross-reactive citrullinated antigens that are also recognized by ACPA [86]. *P. intermedia* has also been shown to induce ACPA formation in RA, but by a different mechanism than *P. gingivalis*, because the serological response to infection with *P. intermedia* was associated with an antibody to a novel citrullinated peptide of cytokeratin 13 (cCK13-1), identified in gingival crevicular fluid, which correlated strongly with anti-tenascin-C (cTNC5) [77]. Since arginine-deiminase activity in RA patients also belonged to *Collinsella* species, this raises the possibility that *Collinsella aerofaciens* may also be responsible for protein citrullination and may contribute to the abnormal autoimmunity in RA [52].

The link between RA and PD is also suggested by the fact that inflamed human periodontal tissue is an extrasynovial source of malondialdehyde-acetaldehyde (MAA)-modified proteins compared to healthy gingival tissue, and at the same time, anti-MAA antibody isotype responses are associated with ACPA in RA patients [87,88]. Malondialdehyde (MDA) is a highly reactive aldehyde produced under conditions of oxidative stress associated with the excessive generation of reactive oxygen species, and MAA adducts are formed when MDA and acetaldehyde react synergistically with proteins [89,90,91]. In addition, in the absence of adjuvants, proteins become highly immunogenic in vivo when haptenized with MAA [92,93,94]. To confirm the role of MAA in the interrelationship between PD and RA, special studies were performed [95]. Serum anti-*P. gingivalis*, anti-*P. intermedia* and anti-*F. nucleatum* antibody levels were found to be positively associated with serum anti-MAA antibody levels in RA patients, confirming an association between these oral pathogens and the generation of anti-MAA antibodies in RA [95].

#### 3.3.2. Production of Immunomodulatory Metabolites

Immunomodulatory properties of bacterial metabolites have also been demonstrated [96]. Altered bacterial metabolism in RA contributes to the development of autoimmunity, while healthy microbiota have an anti-inflammatory effect (Figure 1). For example, *P. gingivalis* DNA, often found in synovial samples from RA patients, contributes to citrullination-independent chronic inflammation and can induce the production of pro-inflammatory cytokines like IL-6, IL-1 and TNF-α, which play a critical role in the onset and progression of RA [97]. The oral administration of *P. gingivalis* was found to significantly aggravate CIA, with increased IL-17 levels in sera and culture supernatants, increased Th17 cell proportions among mesenteric lymphocytes and a significant change in the gut microbiome [98]. In addition, *P. gingivalis* stimulates the gene expression of IFN-γ, the cytokine derived from Th1 cells in the colon [58,99]. *P. gingivalis* can also affect the host’s immune system through an increased lipopolysaccharide (LPS) level [62]. When the intestinal barrier is compromised, LPS, an important component of the outer membrane of Gram-negative bacteria, can enter the systemic circulation in significant amounts, thereby activating immune cells and triggering the production of pro-inflammatory cytokines, leading to inflammation [100]. The abundance of *Collinsella* was found to be correlated strongly with high levels of alpha-aminoadipic acid and asparagine, as well as the production of the pro-inflammatory cytokine IL-17A [48]. *Fusobacterium nucleatum*, which is enriched in patients with RA, was demonstrated to aggravate arthritis in CIA mice through the secretion of outer membrane vesicles (OMVs), which translocate to the joint [101]. It was also shown that *F. nucleatum* OMVs release the virulence factor FadA, which then activates Rab5a-YB1 in synovial macrophages to induce inflammation [101]. Findings from the Phylogenetic Investigation of Communities by Reconstruction of Unobserved States (PICRUSt) analysis also suggest altered amino acid metabolism and proteins involved in lipid biosynthesis, correlated with changed microbiota in RA [52]. The involvement of these alterations in autoantibody formation should be specifically addressed in future studies. The pro-inflammatory activity of tryptophan metabolites has also been shown [102]. Tryptophan is metabolized in three main pathways, indole, kynurenine and serotonin, and the indole pathway is almost exclusively microbiome-derived [102]. Alterations within the indole pathway have been observed in patients with RA, including increased serum indoxyl sulphate and decreased indole-3-acetamide (IAM), indole-3-lactic acid (ILA) and indole-3-aldehyde (I-3aL) [103]. Indole was shown to be significantly elevated in CIA mice, resulting in increased arthritis severity, Th17 cell expansion and pathogenic autoantibody formation [104]. In further experiments, blocking indole production either by reducing dietary tryptophan or by depleting the relevant microbiome protected mice from developing CIA, and supplementing mice with indole caused changes in Th17 production and stimulated the production of pro-inflammatory cytokines such as IL-6, IL-1β and TNF, indicating the development of arthritis [104].

Because of their anti-inflammatory properties, short-chain fatty acids (SCFAs) play an important role in the pathophysiology of RA [105]. SCFAs were important fuels for intestinal epithelial cells (IECs) and regulated IEC functions via multiple pathways to influence their growth, differentiation and roles of subpopulations such as enteroendocrine cells, to affect intestinal motility, and to improve gut barrier functions and to affect metabolism in the host [106]. Under physiological conditions, the bacterial production of SCFAs ensures the normal differentiation of T and B lymphocytes [107]. Numerous studies have demonstrated that SCFAs can decrease the synthesis of pro-inflammatory cytokines, modify gene activity, affect cell proliferation, trigger cell differentiation or apoptosis and modulate immune responses [108]. SCFAs promoted the manufacture of serotonin, which inhibited the development of osteoclasts and bone resorption [109]. *Firmicutes* bacteria are the main producers of butyrate, a member of the SCFA family that plays a central role in the generation and maintenance of Treg cells in the gut, which block the differentiation of T cells into Th17 effectors and Th1 cells and ensure a balanced production of both anti-inflammatory and inflammatory cytokines [110]. *Faecalibacterium prausnitzii* is another representative bacterium that produces butyrate and this microbe exerts anti-inflammatory effects in autoimmune diseases [44]. Butyrate, acetate and propionate administration before the onset of CIA in mice enhanced Bregs frequency and reduced arthritic symptoms, indicating a possible preventative function [111]. Butyrate inhibited the expression of inflammatory cytokines and inhibited autoimmune arthritis through Treg regulation in the CIA mouse model [112]. It was also concluded that butyrate-producing *Subdoligranulum variabile* ameliorates RA, since this bacterium promotes the expression of tumour necrosis factor-inducible gene 6 protein (TSG-6) after co-culturing with joint cells such as HC and HFLS-RA in vitro, along with the down-regulation of the pro-inflammatory cytokine TNF-α in HFLS-RA, a key player in the pathogenesis of RA, thereby reducing RA inflammation [113].

Another metabolite that has a beneficial effect and may help reduce the symptoms of RA is polysaccharide A. Polysaccharide A, secreted by *Bacteroides fragilis*, induces CD4+ T cells to transform into Foxp3+ regulatory T cells (Tregs) that produce IL-10 [114]. The formation of specific bacterial metabolites associated with intestinal dysbiosis has also been shown in another autoimmune disease, SLE, indicating their importance in the pathogenesis of autoimmune diseases, and correcting the composition of metabolites toward those inherent to a healthy body may in the future become one of the ways to treat autoimmune diseases [115].

## 4. RA Management by Manipulating the Gut Microbiota

**Figure 2:**
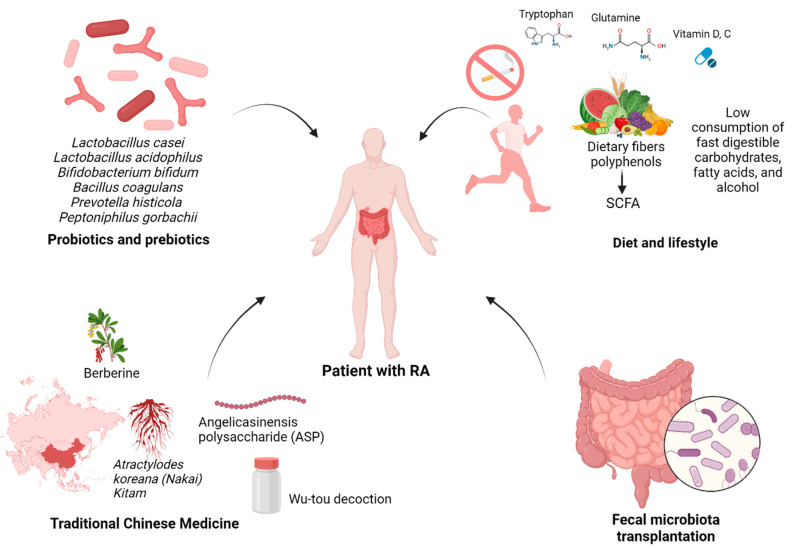
Gut microbiota-targeted strategies for RA therapy. This figure illustrates approaches to modulate gut microbiota and alleviate RA symptoms: (1) probiotics and prebiotics (*Lactobacillus*, *Bifidobacterium*, etc.) to restore microbial balance; (2) diet and lifestyle rich in fibre, polyphenols, vitamins and SCFAs, alongside smoking cessation and reduced intake of harmful foods; (3) traditional Chinese medicine (TCM) (e.g., *Atractylodes koreana* and berberine) to balance microbiota and reduce inflammation; and (4) faecal microbiota transplantation (FMT) to restore microbial diversity and immune homeostasis. These interventions collectively target dysbiosis and systemic inflammation in RA.

Several approaches may be used to correct gut microbiota dysbiosis in the treatment of RA, each with the ultimate goal of shifting the bacterial composition, metabolite profiles and host immune response toward a homeostatic balance that improves the patient’s condition, reduces disease activity and contributes to recovery. The main approaches are using probiotics and prebiotics, special diet, traditional Chinese medicines (TCM) and faecal microbiota transplantation (FMT) (Figure 2). There is also evidence that traditional RA drugs, such as methotrexate and hydroxychloroquine, can restore the diversity of the gut microbiome and reduce pathogenic bacterial species in RA patients [116,117].

### 4.1. Treatment of RA with Probiotics and Prebiotics

The definition of probiotics is “non-pathogenic microorganisms that are able to enter the intestine in sufficient numbers to provide a health benefit to the host” [118]. One possible way involves probiotics aiding the host in maintaining a healthy microbiome, as well as assisting in the restoration of intestinal microbial balance after dysbiosis. Additionally, probiotics have the ability to produce bioactive substances that elicit the desired effects and influence the immune responses of the host [119]. Furthermore, probiotics have been reported to have a beneficial effect on intestinal permeability [120]. Several studies have shown the potential beneficial effects of probiotics in the prevention and treatment of RA [121]. The most studied are the effects of Lactobacillus and Bifidobacterium, which are known to produce anti-inflammatory compounds like short-chain fatty acids (SCFAs) [122]. *Lactobacillus casei* was shown to reduce the pro-inflammatory cytokines IL-12 and TNF-α and to increase anti-inflammatory IL-10 in RA patients [123]. In addition, there was a reduction in RA symptoms in the group of patients on this probiotic [123]. In the CIA model, *L. casei* attenuated the symptoms by reducing pro-inflammatory IL-6 and TNF-α and increasing IL-10 [124]. Concerning Bifidobacterium, it was found that the dysbiosis in the gut microbiota was eliminated, the balance between pro- and anti-inflammatory responses was restored and clinical symptoms were alleviated in CIA rats after administration with five strains of *Bifidobacterium adolescentis* [125]. Another study demonstrated that intervention with *B. longum* RAPO in a preclinical model in CIA mice ameliorated the symptoms of RA, including reduced RA incidence, arthritis score, inflammation, bone damage and cartilage damage [126]. Additionally, B. longum RAPO may play a role in ameliorating RA via inhibiting the secretion of IL-17 and other pro-inflammatory mediators, suggesting its potential role in ameliorating RA [126]. Three bacterial strains have also been found to influence the clinical and metabolic status of RA patients, and the disease activity score on 28 joints (DAS 28) has been observed to be improved after supplementation with *Lactobacillus casei*, *Lactobacillus acidophilus* and *Bifidobacterium bifidum* [127].

*Prevotella* species have also been discovered to have probiotic effects, and data from CIA models suggest that *P. histicola* can suppress antigen-specific Th17 responses, reduce arthritis, increase the transcription of IL-10 and up-regulate antimicrobial peptidase and tight junction proteins [128].

Among the probiotics that have the ability to reduce the symptoms of arthritis, demonstrating immunomodulatory and anti-inflammatory effects, another of the most famous strains is *Bacillus coagulans* [129]. To evaluate the effect of this probiotic, in a double-blind study, 45 patients with RA were administered *Bacillus coagulans* GBI-30, 6086 for 60 days, and the patients showed a significant improvement in the Patient Pain Assessment Score and Pain Scale compared to the placebo group, and they also demonstrated increased mobility [130]. Additionally, *B. coagulans* also generates SCFAs including butyric acid [131]. In an in vivo experiment, arthritic rats were orally administered a combination of probiotic *B. coagulans* and prebiotic inulin to evaluate their possible influence on RA [132]. Treatment results showed significant inhibition of serum amyloid A and fibrinogen production and paw oedema in rats, while a significant reduction in pro-inflammatory cytokines such as TNF-α was observed [132].

*Peptoniphilus gorbachii* (*PG*) was found to produce acetate and butyrate, exhibiting anti-inflammatory properties. In CIA mice, PG administration suppressed arthritis symptoms, reduced the accumulation of inflammatory monocytes in the mesenteric lymph nodes and down-regulated the gene expression of pro-inflammatory cytokines in the ileum. Additionally, *PG* supplementation restored intestinal barrier integrity and partially resolved gut microbial dysbiosis in CIA mice. The faecal microbiota in PG-treated mice corresponded to improved intestinal barrier integrity and reduced inflammatory responses. These data suggest that *PG* holds therapeutic potential for RA by restoring intestinal barrier integrity and suppressing the immunologic response associated with RA [133].

### 4.2. Diet and Lifestyle That Influence the Gut Microbiome in Treating RA

Considerable evidence suggests that appropriate diet and lifestyle can normalize gut microbiota dysbiosis and thereby reduce inflammation and alleviate symptoms in patients with RA. The therapeutic effect is achieved through two possible mechanisms: the restoration of immune tolerance and restoration and maintenance of intestinal barrier function. Diets that contribute to the implementation of this therapeutic effect include the Mediterranean diet, whose characteristics are associated with a high amount of dietary fibre through fruits, vegetables and legumes [134]. In a special study, high-fibre dietary supplementation was observed to increase circulating regulatory T cell numbers, improve Th1/Th17 ratios and decrease markers of bone erosion in RA patients after 28 days of dietary intervention [135]. By stimulating beneficial bacteria that increase SCFA synthesis, dietary fibre also helps improve intestinal barrier function [136]. The beneficial effect is due to the up-regulation of tight junction protein expression by SCFAs [137]. Vitamin D and polyphenols are other important nutrients in the Mediterranean diet that also increase the expression of tight junction proteins [136,138,139]. The amino acids tryptophan and glutamine have been found to reduce bacterial translocation and decrease intestinal inflammation, but the mechanism remains unknown [140,141]. Zinc is also reported to be important in preventing intestinal barrier dysfunction [142]. The therapeutic potential of vitamin C as a modulator of the gut microbiota has also been demonstrated [143]. In this experiment, CIA mice were treated daily with 100 mg/kg vitamin C for 6 weeks, and it was observed that vitamin C effectively corrected the gut microbiota imbalance, specifically reduced the levels of pro-inflammatory cytokines IL-6 and TNF-α, suppressed the inflammatory response and effectively alleviated arthritis symptoms [143].

Some dietary choices are known to have the opposite effect, creating a favourable environment in the gut for the growth of pathogenic bacteria [63]. These properties are found in foods rich in rapidly digestible carbohydrates, fatty acids and alcohol [136,138,144]. In addition, such diets can lead to obesity, which increases intestinal permeability [145]. Patients with obesity have been observed to have elevated levels of *Firmicutes* and *Proteobacteria* as compared to the beneficial species of *Bacteriodetes* [146]. However, this condition can be corrected, and clinical studies have shown that increased intestinal permeability in obese patients decreases after weight loss [145]. One of the bacterial species, *Akkermansia muciniphila*, has been found to improve intestinal barrier function and control diet-induced obesity after daily administration [147,148].

But moderate alcohol consumption has been found to be a protective factor in the pathogenesis of RA [149,150]. To clarify the mechanism of alcohol influence, in a special study, CIA mice were given drinking water containing 10% (*v*/*v*) ethanol, and the results showed that Muribaculaceae was predominant in the gut microbiota of mice after ethanol treatment, and the levels of the microbiota metabolite acetate were increased [151]. Acetate has been shown to reduce arthritis by a reduction in neutrophils and serum myeloperoxidase-deoxyribonucleic acid complex [151].

An important component of a healthy lifestyle is not smoking, especially since tobacco smoking has been shown to affect the composition of the gut microbiota [152]. The results confirmed the effects of smoking on three bacterial taxa, *Intestinimonas*, *Catenibacterium* and *Ruminococcaceae,* and identified another 13 taxa which may be causally affected by tobacco smoking. A positive role in smoking cessation associated with *Actinobacteria* has been shown, and the mechanism of the association between parental smoking and the early initiation of smoking in their children appears to be determined by *Bifidobacteria* [152].

### 4.3. Traditional Chinese Medicine (TCM) for RA

TCM is also able to regulate the gut microbiota of RA patients. TCM may regulate the production of SCFAs by acting on the gut microbiota, thereby affecting the disease [153]. After the treatment of CIA rats with Atractylodes koreana (Nakai) Kitam, the ratio of *Firmicutes*/*Bacteroides* increased and *Proteobacteria* and *Verrucomibia* decreased and the inflammatory cytokines like TNF-α, IL-1 and IL-1β in the plasma of CIA rats also decreased [154]. The therapeutic mechanism was suggested to be related to the improvement of SCFA imbalance in addition to the down-regulation of inflammatory factors [154]. Another compound, berberine, can reduce the diversity and abundance of intestinal bacteria in CIA rats, but can increase the diversity of butyrate-producing bacteria, significantly increase the level of intestinal butyrate and promote the production of butyrate by regulating gut microbiota as a therapeutic agent for RA [155]. In addition to acting on SCFAs, TCM can also improve other amino acid metabolism through the gut microbiota [153]. A wu-tou decoction can partially inhibit inflammation and regulate gut barrier function by adjusting *Bacteroides, Prevotella*, and *Akkermansia* and their relation to SCFAs, cholic acid and indole propionic acid to improve RA [156]. Paeonia glycosides intervention increased the relative abundance of beneficial symbiotic bacteria *Ruminococcacea*, *Oscillabacter* and *Paraactoides* in CIA rats, down-regulated the levels of Th1 cells and Th17 cells in CIA rats and up-regulated the levels of Th2 cells and Treg cells [157]. 16S rDNA sequencing suggested that Angelica sinensis polysaccharide (ASP) could shape the gut microbiota composition [158]. The colonic transcriptome showed that ASP regulates Cldn5 to improve intestinal dysfunction induced by RA, and regulates the expression of Slit3 and rgs18 to regulate the balance of osteoblasts and osteoclasts, which may be related to gut microbiota [158].

### 4.4. Faecal Microbiota Transplantation (FMT) for RA

One of the most effective approaches to successfully restore the dysbiosis of the intestinal microbiota is FMT. FMT is the injection of a faecal suspension obtained from a healthy donor into the patient’s gastrointestinal tract to normalize the balance of the intestinal microbiota [159]. The following putative mechanisms can be identified for the therapeutic effect of FMT. The application of a donor faecal suspension restores the composition and diversity of the intestinal microbiota, improves local and systemic immune homeostasis, enhances intestinal barrier function, reduces mucosal inflammation, and increases levels of microbiota-derived metabolites such as SCFAs [160]. The therapeutic potential of FMT was first studied in intestinal infections, and this procedure was added to the standard treatment recommendations for *Clostridium difficile* infection [161]. Clinical trials of FMT have already been conducted in patients with other autoimmune diseases, such as type 1 diabetes and ulcerative colitis, with some success [162,163]. The efficacy of FMT has also been demonstrated in a mouse model of SLE [164,165]. This procedure was then tested on a complex SLE patient infected with the parasite Blastocystis hominis, who also had glomerulonephritis, malnutrition, diarrhoea and severe weight loss, and the treatment resulted in a significant improvement in overall condition and a reduction in all of these symptoms [166]. The following 20 patients with active SLE were recruited to participate in a pilot clinical study that successfully confirmed the safety and efficacy of the FMT procedure [167].

All of these data raised hopes that RA patients could also be successfully treated with FMT, and such a procedure was recently performed on an RA patient [168]. The donor faecal microbiota was introduced into the patient’s colon via colonoscopy under anaesthesia, resulting in a significant reduction in the Health Assessment Questionnaire Disability Index (HAQ-DI) from 0.7 to 0.05 on day 7 after the procedure, which was maintained for at least 4 months, and a reduction in the DAS28 index [168]. This study leaves a number of questions that need to be answered in further clinical trials with a larger number of RA patients. It is necessary to find out what the optimal dose is, how long the therapeutic effect lasts, how often the procedure needs to be performed, and most importantly, how safe the procedure is. However, initial data suggest that the procedure is effective and safe. One of the clinical trials investigating the efficacy of FMT in RA is now in Phase II (NCT03944096). Another is currently recruiting (NCT05790356).

Experience has shown that the quality of the donor material is critical to the safety and efficacy of the procedure. Patients with autoimmune diseases, including RA, are thought to be more susceptible to latent pathogens due to the long-term use of immunosuppressive drugs, which poses unknown risks. To prevent the transmission of pathogenic bacteria, viruses and fungi from the donor to the recipient, the donor undergoes a series of medical examinations, including a detailed medical history and blood and stool tests [160]. Donors with infectious diseases, autoimmune diseases, gastrointestinal diseases, metabolic syndrome, or surgery or those taking antibiotics or immunosuppressants are not eligible [169]. The screening of the recipient is no less important. General contraindications include pregnancy, infectious diseases, recent gastrointestinal surgery, anticoagulant therapy and the use of antibiotics or probiotics [160].

## 5. Conclusions and Future Perspectives

Despite significant advances in the treatment of RA in recent years, some patients remain refractory to clinically used drugs and are at a high risk of serious complications and even death. In addition, some medical treatments cause side effects and are very expensive. There is a need to develop alternative treatments that are inexpensive, safe and effective. Numerous data indicate that the gut microbiota influences almost all biological processes in the host, and that microbiota dysbiosis is associated with impaired immune tolerance and the development of RA. Several mechanisms have been proposed to explain the relationship between gut microbiota dysbiosis and the pathogenesis of RA, such as bacterial translocation due to increased intestinal permeability, molecular mimicry and the production of bacterial metabolites that may lead to citrullination or have immunomodulatory properties by inducing inflammation. However, pathological conditions associated with intestinal dysbiosis may be reversible. The main methods of correcting intestinal dysbiosis proposed for use in patients with RA are the use of probiotics/prebiotics, a special diet, and FMT. TCM preparations also have therapeutic potential.

Targeting the gut microbiota offers a considerable potential for an effective RA therapy, especially because it does not require expensive drugs or equipment, but only the mobilization of the body’s internal resources. Probiotics/prebiotics demonstrated their ability to modulate the gut microbiota, reduce inflammation and alleviate symptoms in animal models of arthritis and in patients. Despite the various challenges that remain in probiotic research, such as determining targets, pathways and mechanisms, the numerous benefits they offer to the human body continue to drive researchers in their quest for further exploration. Diet is one of the most important environmental factors triggering RA. Through nutrition, the gut microbiome can be affected, which allows for the improvement of intestinal homeostasis. FMT performed in one patient has shown preliminary efficacy and safety, but additional data from clinical trials with larger numbers of patients are needed.

Unfortunately, the association between gut dysbiosis and immune dysregulation in RA has not been established; it is not clear whether gut microbiome dysbiosis is a consequence of the disease or a driving factor in its pathogenesis. In the future, this aspect needs to be considered in order to facilitate the development of new ways to influence the gut microbiota and the search for new drugs that can effectively influence the microbiota. Future research should identify the causes of dysbiosis and determine exactly how and when gut dysbiosis influences the development of RA. Considering the amount of knowledge and experience available, there is no doubt that in the near future, methods of treating RA based on the manipulation of the gut microbiota will enter clinical practice, at least as an adjuvant treatment in addition to those already available, and will allow us to take RA therapy to a new level—a level that will significantly improve the quality of life of patients.

## Figures and Tables

**Table 1:** Gut microbiota and cytokine alterations in RA. This table highlights changes in microbial composition and cytokine levels observed in RA patients and murine models, emphasizing the role of dysbiosis and inflammation in disease progression. Key findings include increased pro-inflammatory bacteria, reduced microbial diversity, and elevated cytokines like IL-6, IL-17 and TNF-α.

Object of Study	Healthy Control	Bacteria		Cytokine Levels	Additional Info	Ref.
		Increased	Decreased			
Murine models						
CIA mice	+	*Desulfovibrio* *Prevotella* *Parabacteroides* *Odoribacter* *Acetatifactor* *Blautia* *Coprococcus* *Ruminococcus*	*Enterorhabdus* *Myroides* *Rikenella* *Brochothrix* *Lactococcu* *Streptococcus*		Antibiotic treatment worsened arthritis and increased levels of IL-6, IFN-γ and IL-17	[45]
CIA mice		*Ruminococcaceae* *Lachnospiraceae* *Desulfovibrinocaceae*	S24-7 *Bacteroidaceae*	IL-17 ↑ GM-CSF ↑ TNF-α ↑ IL-6 ↑ IL-8 ↑	Elimination of the gut microbiota by antibiotics during established arthritis reduced gut Th17 cells and attenuated arthritis	[46]
Human study						
RA patients	+	*Actinobacteria Collinsella* *Eggerthella* *Turicibacter* *Streptococcus*	*Faecalibacterium*	IL-17A ↑	Decreased bacterial diversity	[48]
RA patients with positive anti-CCP antibodies	+	*Verrucomicrobiae* *Blautia* *Akkermansia* *Clostridiales*			Lower diversity index of gut microbiota	[49]
High TNF-α and IL-17A levels		*Enterobacteriaceae* *Klebsiella*	*Bifidobacterium*	TNF-α ↑ IL-17A ↑		
ACPA-positive patients		*Blautia* *Akkermansia* *Clostridiales*				
RA patients		*Prevotella denticola*				[50]
RA patients	+	*Collinsella* *Bifidobacterium* *Enterococcus* *Sedimentibacter*	*Dorea* *Sarcina*	IL-1β ↑ IL-6 ↑ TNF-α ↑	A number of metabolic pathways, including fatty acid biosynthesis and glycosaminoglycan degradation, were enriched in the case–control study	[51]
RA patients	+	*Collinsella aerofaciens* *Sedimentibacter* *Enterococcus*	*Sarcina* *02d06* *Porphyromonas* *Dorea formicigenerans*	IL-17 ↑ IL-17A ↑	Create an inflammatory imbalance and induce Th17 or Th1 responses	[52]
RA patients		*Enterococci* *Clostridia* *Enterobacteria* *Staphylococci*	*Lactobacteria* *Bifidobacteria* *Bacteroids* *Lactopositive colibacteria*			[53]
Patients up to 6 months after the diagnosis	+	*Prevotella*	*Bacteroides*	IL-17 ↑	Molecular mimicry—molecular structures of a *Prevotella copri* resemble those of the host	[54]
Newly diagnosed RA with anti-CCP antibodies	+	*Lachnospiraceae* *Helicobacteraceae* *Ruminococcaceae* *Erysipelotrichaceae* *Bifidobacteriaceae*				[55]
Faecal, dental and salivary samples from RA patients	+	*Lactobacillus salivarius*	*Haemophilus* spp.			[56]
Both RF and ACPA positive			*Haemophilus* spp.		Similar findings were observed in their dental and salivary samples	
Higher inflammatory protein levels		*Lactobacillus salivarius*	*Haemophilus* spp.			
